# Viral communities associated with porcine respiratory disease complex in intensive commercial farms in Sichuan province, China

**DOI:** 10.1038/s41598-018-31554-8

**Published:** 2018-09-06

**Authors:** Sinan Qin, Wenqiang Ruan, Hua Yue, Cheng Tang, Kelei Zhou, Bin Zhang

**Affiliations:** 1 College of Life Science and Technology, Southwest Minzu University, Chengdu, 610041 China; 2 Key laboratory of Ministry of Education and Sichuan Province for Qinghai-Tibetan Plateau Animal Genetic Resource Reservation and Utilization, Chengdu, 610041 China; 3 Animal Disease Prevention and Control Innovation Team in the Qinghai-Tibetan Plateau of State Ethnic Affairs Commission, Chengdu, 610041 China

## Abstract

Porcine respiratory disease complex (PRDC), a common piglet disease, causes substantive economic losses in pig farming. To investigate the viral diversity associated with PRDC, the viral communities in serum and nasal swabs from 26 PRDC-affected piglets were investigated using metagenomics. By deep sequencing and *de novo* assembly, 17 viruses were identified in two pooled libraries (16 viruses from serum, nine from nasal swabs). Porcine circovirus (PCV)-2, porcine reproductive and respiratory syndrome virus (PRRSV) and pseudorabies virus, all commonly associated with PRDC, were identified in the two pooled samples by metagenomics, but most viruses comprised small linear and circular DNAs (e.g. parvoviruses, bocaviruses and circoviruses). PCR was used to compare the detection rates of each virus in the serum samples from 36 PRDC-affected piglets versus 38 location-matched clinically healthy controls. The average virus category per sample was 6.81 for the PRDC-affected piglets and 4.09 for the controls. Single or co-infections with PCV-2 or PRRSV had very high detection rates in the PRDC-affected piglets. Interestingly, porcine parvovirus (PPV)-2, PPV-3, PPV-6 and torque teno sus virus 1a were significantly associated with PRDC. These results illustrate the complexity of viral communities in the PRDC-affected piglets and highlight the candidate viruses associated with it.

## Introduction

Porcine respiratory disease complex (PRDC), which causes major losses in the pig farming industry, is characterized by retarded growth performance, increased mortality and antimicrobial use in PRDC-affected pigs, and extra expenditure on control measures against it^1^. PRDC is multifactorial in origin, with both infectious and non-infectious factors contributing to the respiratory disease it causes, which is predominantly seen in pigs of 1 to 3 months of age^2,3^. The onset of respiratory disease on piglet farms is thought to be related to the initial stress of piglet transportation to the farms, after which a primary viral insult causes a secondary infection from the bacteria resident in these animals^1,4^. Hence, based on their ability to damage the upper airway epithelium, injure the lung parenchyma and promote secondary bacterial colonization, the primary viral pathogens are able to influence the development and outcome of PRDC^5^.

Porcine reproductive and respiratory syndrome virus (PRRSV), porcine circovirus type 2 (PCV-2) and swine influenza virus (SIV) are frequently identified as the primary pathogens associated with PRDC, a disease that can lead to severe respiratory distress in pigs^1,5,6^. Genetic recombination and mutation are the most common generators of genetic diversity in organisms and the emergence of novel PRRSV, PCV-2 and SIV strains increases the difficulty of clinical diagnosis and disease control against such viruses^7–9^. Several novel viruses have been discovered in diseased piglets with PRDC, including novel parvoviruses, bocaviruses and torque teno sus virus (TTSuV)^10–14^. However, the mechanism underlying the pathogenesis of PRDC remains unclear. To complicate matters further, the viral pathogens associated with respiratory diseases in pigs vary significantly among farms, production sites, regions and countries, making generalisations about how to treat and control PRDC difficult^1^. Hence, establishing accurate diagnostic criteria for PRDC is critical for implementing the most appropriate treatment and control regimens against it on farms.

As a high-throughput sequencing technology, viral metagenomics has been shown to be a powerful tool for identifying large numbers of known and novel viruses, and for investigating the viromes associated with complex disease syndromes^15,16^. Metagenomic sequencing has also been used to discover and characterize the viruses associated with respiratory diseases in humans, dairy and feedlot cattle^17–19^. However, on intensive commercial farms, the viral communities associated with PRDC are largely unknown. Therefore, in the present study, we utilized viral metagenomic sequencing to characterize the viromes from serum and nasal swabs collected from piglets with acute PRDC in Sichuan province, China. We also examined the detection rates for viruses identified in animals with PRDC and location-matched the rates with clinically healthy controls to determine which respiratory viruses are associated with PRDC.

## Results

### Serum viromes

**Figure 1:**
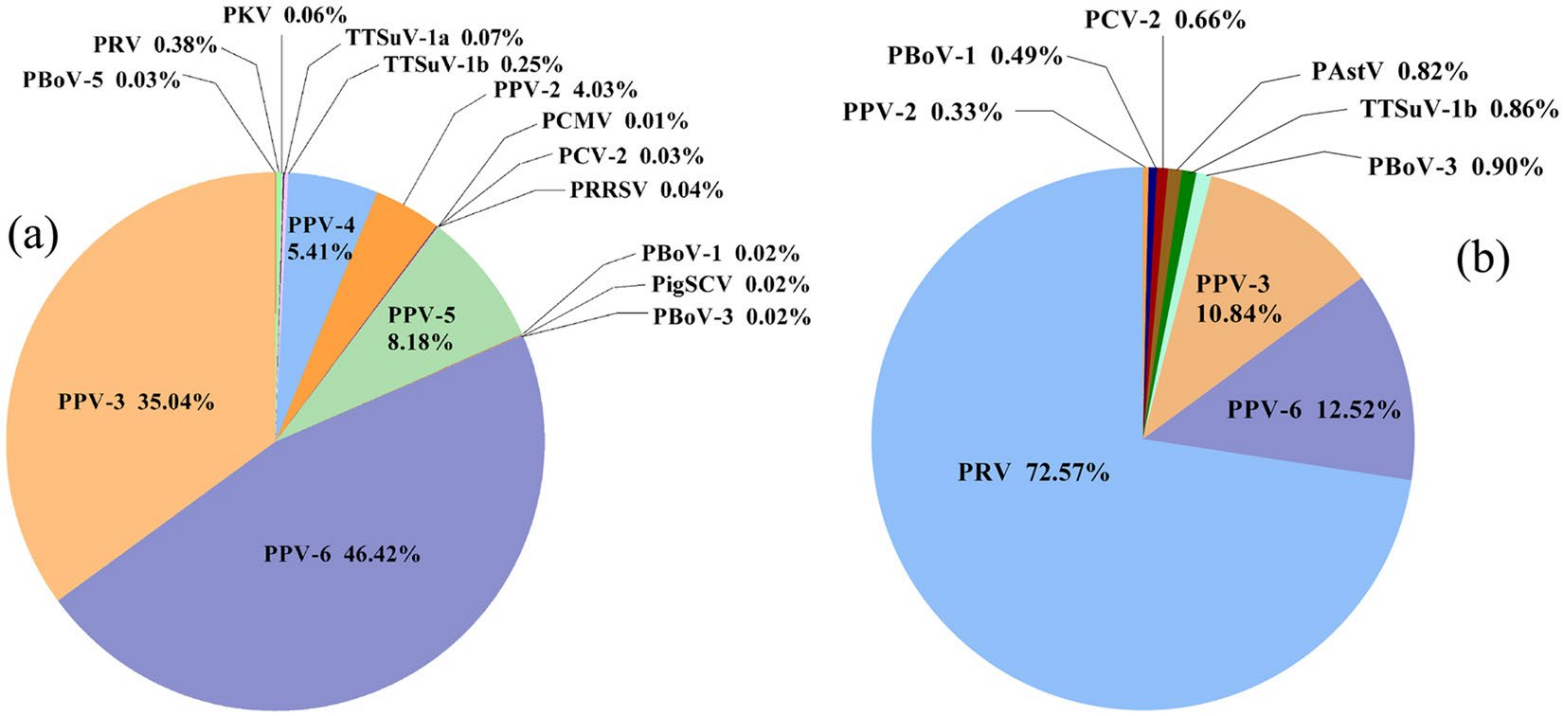
Sequence classifications and percentages of the viruses detected in the pooled serum samples (**a**) and nasal swabs (**b**) from the 26 PRCD-affected piglets.

Twenty-six serum samples from PRDC-affected piglets were pooled and then sequenced using the Illumina 4000 system. We obtained approximately 10 million reads in total from the serum samples (GenBank accession number: SRX2901706). Reference genomes were downloaded from GenBank to screen for the best BLAST hits and the raw sequencing reads were mapped to them using the template assemblies. Assemblies were inspected manually and only where one unique read as a minimum mapped to multiple regions of the viral genome was the virus considered to have been detected, and the number of reads was tallied. Sixteen distinct viruses were identified in the serum samples from the diseased piglets (Fig. 1a), which, in order of sequence read abundance, are as follows: porcine parvovirus (PPV) 6 (PPV-6, 46.42% of all reads), PPV-3 (35.04%), PPV-5 (8.18%), PPV-4 (5.41%), PPV-2 (4.03%), porcine pseudorabies virus (PRV, 0.38%), torque teno sus virus 1b (TTSuV-1b, 0.25%), TTSuV-1a (0.07%), porcine kobuvirus (PKV, 0.06%), porcine reproductive and respiratory syndrome virus (PRRSV, 0.04%), porcine circovirus 2 (PCV-2, 0.03%), porcine bocavirus 5 (PBoV-5; 0.03%), ungulate bocaparvovirus 2 (PBoV-1, 0.02%), ungulate bocaparvovirus 5 (PBoV-3, 0.02%), pig stool associated circular ssDNA virus (PigSCV, 0.02%) and porcine cytomegalovirus (PCMV, 0.01%).

### Nasal swab viromes

We obtained approximately 19.7 million reads from the nasal swabs (GenBank accession number: SRX2901558). Approximately 0.1% of the sequence reads mapping to mammalian viral sequences were recovered from these swabs. Nine distinct mammalian viruses were identified from the swabs, as determined by the sequence reads from each virus (Fig. 1b). The viruses, in order of sequence read abundance, were PRV (72.57% of all reads), PPV-6(12.52%), PPV-3 (10.84%), PBoV-3 (0.9%), TTSuV-1b (0.86%), porcine astrovirus (PAstV, 0.82%), PCV2 (0.66%), PBoV-1 (0.49%) and PPV-2 (0.33%). These results show that at least 17 different viruses were identifiable in the libraries prepared from the serum and nasal swab samples from the piglets, and that the viral communities are complex and diverse in the piglets with PRDC.

### Viral genome assembly

BLASTN analyses revealed that the sequence reads from the serum and nasal swab libraries matched 17 mammalian viruses known to cause infective respiratory diseases in pigs. The contigs from these viruses were *de novo* assembled using SOAP assembly software, from which six complete or near full-length genome sequences (PPV-2, PPV-3, PPV-4, PPV-5, PPV-6, and PCV-2), were assembled using the corresponding viral sequence contigs (Table 1).

**Figure 2:**
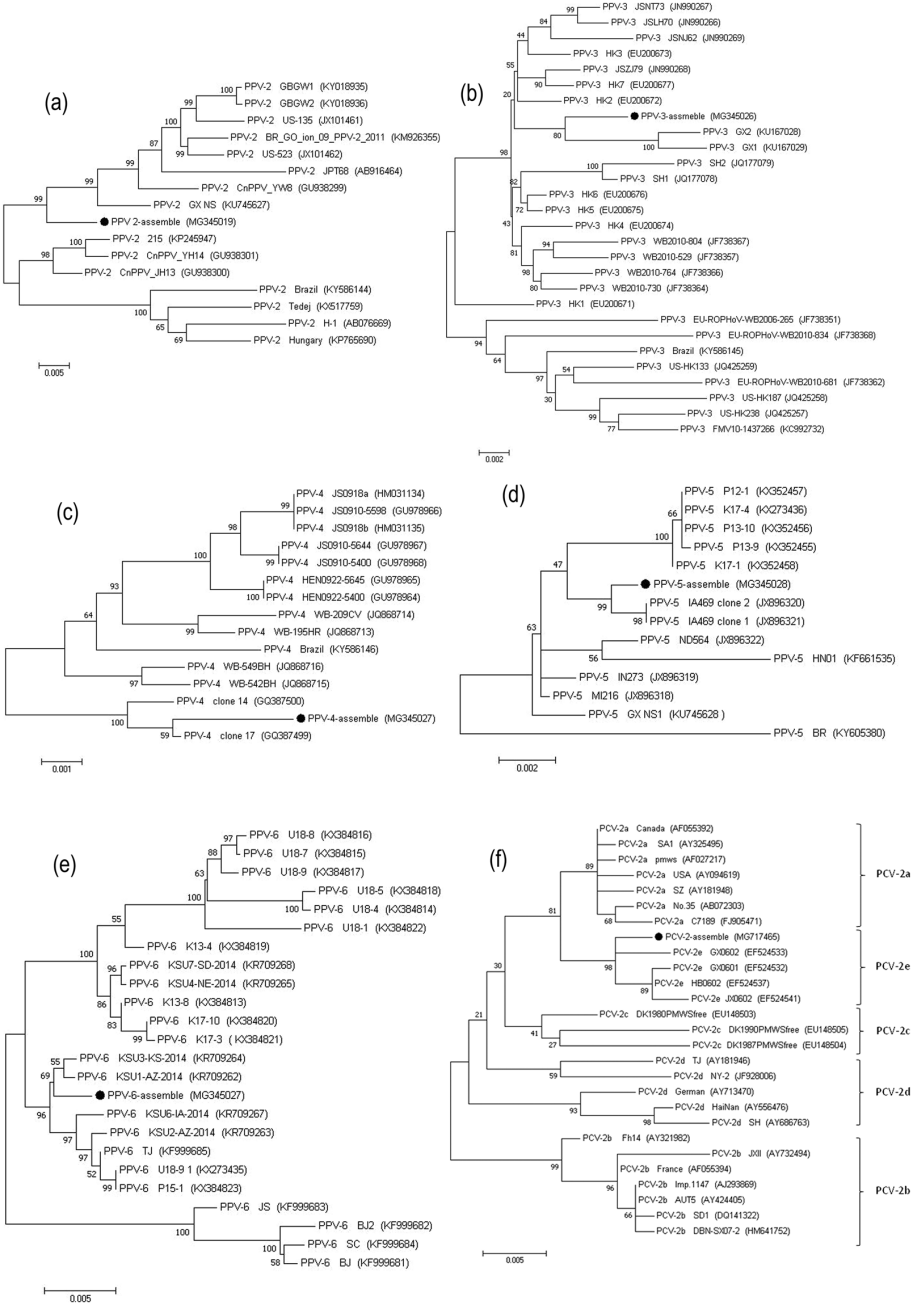
Phylogenetic analyses of six complete or near-complete viral genome sequences: PPV-2 (**a**), PPV-3 (**b**), PPV-4 (**c**), PPV-5 (**d**), PPV-6 (**e**), PCV-2 (**f**). Phylogenetic trees were constructed with MEGA 7.0 software by the maximum-likelihood method with 1,000 bootstrap replicate values. The viral assembly sequences characterized in this study are marked by black circles. GenBank accession numbers for the viral reference strains are shown in parentheses.
(ii) **PPV-3**. Deep sequencing, which provided 100% coverage of the total genome length for PPV-3, generated 13,554 sequence reads related to this virus. The full-genome sequence assembled for the PPV-3 isolates revealed that the PPV-3 genome is 5,081 bp in length, with a GC content of 51.56%. The virus shares 96.8% to 99.1% nucleotide sequence similarity with 27 GenBank PPV-3 reference sequences. The phylogenetic tree constructed for the PPV-3 isolates and the 27 PPV-3 GenBank genome sequences (Fig. 2b) revealed that PPV-3 is closely related to GX1 and GX2, two novel PPV-3 isolates identified in tonsil samples from piglets in China^20^.
(iii) **PPV-4**. PPV-4 was identified via 2,052 sequence reads. The complete viral genome sequence we assembled was 5,908 bp in length, with a GC content of 42.5%. The assembled PPV-4 virus shares 98.1% to 99.6% sequence identity with 14 PPV-4 reference sequences from GenBank. In the phylogenetic tree, the PPV-4 isolates evidently share close genetic relationships with the clone 17 strain from the USA (Fig. 2c)^21^.
(iv) **PPV-5**. PPV-5 was identified via 3,100 sequence reads by deep sequencing of the sera samples. The complete genome sequence (97.2%) for the PPV-5 isolates, which was assembled from a 5,458 bp sequence (Table 1), shares high sequence similarities (97.0% to 99.5%) with 13 PPV-5 GenBank reference strains. Further analysis of the phylogenetic tree indicated that the PPV-5 isolates share close relationships with both of the USA-isolated clone 1 and 2 IA469 strains^22^ (Fig. 2d).
(v) **PPV-6**. Analysis of 17,906 sequence reads showed that they were closely related to PPV-6 genomic sequences. The complete genome sequence we obtained for the PPV-6 isolates (Table 1) is 6,199 bp in length, with a GC content of 46.87%. It shares a high level of sequence identity (97.4% to 99.6%) with 23 PPV-6 GenBank reference strains. A phylogenetic tree was constructed to assess the genetic evolutionary aspects of the assembled PPV-6 isolates with respect to the 23 GenBank PPV-6 isolates (Fig. 2e). The results show that the virus shares close genetic relationships with KSU3-KS-2014 and KSU1-AZ-2014 strains, which were first identified in 2015 in PRRSV-positive piglet serum in the USA^10^.
(vi) **PCV-2**. We identified 28 sequences that are closely related to PCV-2 in the serum samples and nasal swabs by deep sequencing. The 1,310 bp sequence we assembled covers 74.1% of the total genome length (Table 1), and encodes the capsid protein and a partial replicase protein. The partial PCV-2 genome shares 95.9% to 99.4% sequence identity with the 35 reference PCV-2 genomes in GenBank. Based on a sequence motif in the capsid protein, five genotypes (a, b, c, d, and e) have been described for PCV-2^23^. The phylogenetic tree we constructed to discern the evolutionary relationships between the assembled PCV-2 sequence and the 35 other genotypically distinct PCV-2 reference strains showed that although the assembled virus fell into the PCV-2e group, it was located in a unique cluster (Fig. 2f).

(i) **PPV-2**. A total of 1,535 reads with sequence identities corresponding to PPV-2 were detected in the libraries prepared from the serum and nasal swabs. A near complete genome sequence of 5,438 bp long and covering 98.9% of the whole genome sequence was assembled from the PPV-2 isolates (Table 1). The sequence identity scores for PPV-2 were 94.8% to 97.3% when compared with 15 PPV-2 GenBank reference sequences. Phylogenetic analysis indicated that the assembled PPV-2 isolates have distant genetic relationships with the 15 other PPV-2 viruses (Fig. 2a), and are located in a unique cluster. This result suggests that the PPV-2 virus identified herein has a novel genotype.

**Table 1:** Virus assembly from serum- and nasal-swab libraries using deep sequencing.

Virus	Family	GenBank acceession no.	Conting length (bp)	No. ofsequence reads	Coverage of total genome length (%)
Porcine parvovirus 2	Parvovirinae	MG345019	5,438	1,527	98.9
Porcine parvovirus 3	Parvovirinae	MG345026	5,081	13,290	100.0
Porcine parvovirus 4	Parvovirinae	MG345027	5,908	2,052	100.0
Porcine parvovirus 5	Parvovirinae	MG345028	5,458	3,100	97.2
Porcine parvovirus 6	Parvovirinae	MG345036	6,199	17,601	100.0
Porcine circovirus 2	Circoviridae	MG717465	1,310	16	74.1

### Virus detection in lung samples from the PRDC-affected piglets

Lungs from the 26 PRDC-positive piglets were used to PCR verify the detection rates of the 17 different viruses. From these 26 samples (Table 2), PCV-2 was identified in 96.2% (25/26), PRRSV in 84.6% (22/26), PAstV in 84.6% (22/26), PPV-2 in 73.1% (19/26), PKV in 69.2% (18/26), PCMV in 57.7% (15/26), PPV-3 in 53.8% (14/26), PBoV-1 in 46.2% (12/26), TTSuV-1b in 42.3% (11/26), PPV-5 in 34.6% (9/26), PPV-6 in 34.6% (9/26), TTSuV-1a in 19.2% (5/26), PBoV-3 in 15.4% (4/26), PRV in 7.69% (2/26), PBoV-5 in 7.69% (2/26), PigSCV in 7.69% (2/26) and PPV-4 in 3.85% (1/26). In the individual lung samples, the mean number of virus categories for each sample (co-infections) was 7.4 for piglets with PRDC, with a maximum of 11 different viruses shed in one sample. The above results indicate that co-infections with multiple viruses were common in the diseased piglets with PRDC. According to the PCR test results, we found that PCV-2 and PRRSV were the viruses most frequently detected in the individual lung samples from the PRDC-affected piglets.

**Table 2:** Viruses detected by PCR in 26 lung samples from piglets with PRDC.

Virus	Sample																										Rate^*^
	S1	S2	S3	S4	S5	S6	S7	S8	S9	S10	S11	S12	S13	S14	S15	S16	S17	S18	S19	S20	S21	S22	S23	S24	S25	S26	
PCV-2	+	+	+	+	+	+	+	+	+	+		+	+	+	+	+	+	+	+	+	+	+	+	+	+	+	25/26
PRRSV		+^a,b^	+^b^	+^b^	+^a,b^	+^a,b^	+^a,b^	+^a,b^	+^a,b^	+^a,b^	+^a,b^	+^a,b^	+^b^		+^a^	+^a^	+^a,b^			+^1^	+^b^	+^b^	+^a^	+^b^	+^b,c^	+^b^	22/26
PRV																			+				+				2/26
PAstV	+	+	+	+	+	+	+	+	+	+	+	+	+	+	+	+		+	+		+			+	+	+	22/26
PPV-2	+	+		+	+			+	+					+	+	+	+	+	+	+	+	+	+	+	+	+	19/26
PKV	+	+	+	+	+	+	+	+	+	+	+	+	+		+		+	+				+	+				18/26
PCMV	+	+	+	+			+	+		+		+		+	+	+	+			+		+		+			15/26
PPV-3				+	+			+	+	+	+			+			+	+	+	+			+	+		+	14/26
PBoV-1						+	+			+			+	+	+		+	+				+	+	+		+	12/26
TTSuV-1b	+	+		+										+	+	+				+	+	+	+		+		11/26
PPV-5	+	+											+	+	+	+			+		+		+				9/26
PPV-6	+	+			+					+				+						+		+		+		+	9/26
TTSuV-1a	+													+	+	+		+									5/26
PBoV-3																+			+			+		+			4/26
PBoV-5												+							+								2/26
pigSCV											+													+			2/26
PPV-4														+													1/26
No. of viruses	9	9	5	8	7	5	6	7	6	8	5	6	6	11	10	9	7	7	8	7	6	9	9	10	5	7	

Sample S1 and S2 were from the same farm, Sample S3 and S4 were from the same farm, Sample S5 and S6 were from the same farm, Sample S7 and S8 were from the same farm, Sample S9 and S10 were from the same farm, Sample S11–S13 were from the same farm, Sample S14–S16 were from the same farm, Sample S17–S19 were from the same farm, Sample S20–S22 were from the same farm, Sample S23 and S24 were from the same farm, Sample S25 and S26 were from the same farm.

^*^Detection rates of the17 different viruses in the 26 lung samples from PRDC.

^a^Highly pathogenic PRRSV; ^b^NADC 30-like PRRSV; ^c^Classical PRRSV.

In China, the epidemiology of PRRSV is complicated, and three major PRRSV genotypes are co-prevalent on pig farms: classical PRRSV, highly pathogenic (HP) PRRSV and NADC30-like PRRSV^24,25^. Here, 22 positive samples were detected in 26 piglets with PRDC. These three major genotypes were detected in the PRRSV-positive samples; specifically, NADC30-like PRRSV in 69.2% (18/26) of the samples, HP-PRRSV in 50% (13/26) of the samples and classical-PRRSV in 3.8% (1/26) of the samples. Thus, NADC30-like PRRSV was the main strain circulating in China, a result consistent with some reports on the molecular epidemiology of the NADC30-like strain in recent years^25,26^. Furthermore, co-infections with different PRRSV genotypes were highly prevalent in piglets with PRDC, and the most frequent co-infections involved NADC30-like PRRSV and HP-PRRSV(10/26), which shows that the prevalence of PRRSVs is more complex and diverse in piglets with PRDC in Sichuan province, China.

### Virus detection rate in serum from PRDC cases versus controls

To better understand which viruses are associated with PRDC, the previously determined 17-virus detection rate was investigated in an additional 74 serum samples from 36 PRDC-affected piglets and 38 location-matched clinically healthy control animals between 1 to 2 months of age, which were collected from five farms in 2017. The results of the PCR-based, case-control study are shown in Tables 3 and S2. With the individual serum samples, on average, 6.81 different viruses were shed by the 36 PRDC-affected piglets, revealing a high prevalence of viral co-infections in these respiratory-diseased piglets. In contrast, the mean value for the 38 clinically healthy controls showed that 4.09 distinct viruses were shed into the serum samples. A statistically greater number of piglets with PRDC shed 6 or more viruses (27/36) compared with the clinically healthy controls (11/38) (OR = 7.3, OR 95% CI = 2.63–20.6, P <  0.001).

**Table 3:** Detection rates of 17 viruses in 74 serum samples from PRDC-affected piglets and clinically healthy controls.

Virus	No. positive/total		OR	OR 95% CI	*P* value
	Asymptomatic samples	Animals with PRDC			
PCV-2	44.74% (17/38)	72.22% (26/36)	3.22	1.22–8.47	0.01
PRRSV	31.58% (12/38)	75.0% (27/36)	6.50	2.34–18.0	<0.001
PRV	2.63% (1/38)	2.78% (1/36)	1.48	0.09–24.78	0.65
PPV-2	52.63% (20/38)	83.33% (30/36)	4.50	1.52–13.30	0.005
PPV-3	13.16% (5/38)	52.78% (19/36)	7.38	2.35–23.20	<0.001
PPV-4	7.89% (3/38)	2.78% (1/36)	0.33	0.033–3.36	0.33
PPV-5	7.89% (3/38)	5.56% (2/36)	0.68	0.11–4.37	0.53
PPV-6	36.84% (14/38)	83.33% (30/36)	8.57	2.86–25.67	< 0.001
PBoV-1	7.89% (3/38)	19.44% (7/36)	2.81	0.67–11.88	0.13
PBoV-3	10.53% (4/38)	8.33% (3/36)	0.77	0.16–3.72	0.53
PBoV-5	7.89% (3/38)	5.56% (2/36)	0.69	0.11–4.37	0.53
TTSuV-1a	1.84% (7/38)	47.22% (17/36)	3.96	1.39–11.31	0.008
TTSuV-1b	36.84% (14/38)	58.33% (21/36)	2.40	0.94–6.11	0.052
PCMV	21.05% (8/38)	44.44% (16/36)	3.00	1.08–8.32	0.03
PKV	13.16% (5/38)	16.67% (6/36)	1.32	0.36–4.77	0.46
PAstV	63.16% (24/38)	83.33% (30/36)	2.92	0.97–8.73	0.051
pigSCV	5.26% (2/38)	11.11% (4/36)	2.25	0.39–13.12	0.31

As PRDC-associated pathogens, both PCV-2 (OR = 3.22, OR 95% CI = 1.22–8.47, *P* = 0.01) and PRRSV (OR = 6.5, OR 95% CI = 2.34–18.0, *P* < 0.001) had statistically significant different detection rates in the serum samples between the PRDC cases and controls. Also, a significant difference (OR = 4.95, OR 95% CI = 1.73–14.13, *P* = 0.002) in the number of co-infections with PCV-2 and PRRSV was observed in the serum samples from the PRDC cases compared with the clinically healthy controls. Interestingly, several novel viruses had significantly different detection rates in the sera from the PRDC-affected piglets compared with the clinically healthy animals; these included PPV-2 (OR = 4.5, OR 95% CI = 1.52–13.3, *P* = 0.005), PPV-3 (OR = 7.38, OR 95% CI = 2.35–23.2, *P* < 0.001), PPV-6 (OR = 8.57, OR 95% CI = 2.86–25.67, *P* < 0.001) and TTSuV-1a (OR = 3.96, OR 95% CI = 1.39–11.31, *P* = 0.008). In contrast, no significant differences were observed for the PPV-4, PPV-5, PBoV-1, PBoV-3, PBoV-5, TTSuV-1b, PCMV, PKV, PAstV and pigSCV detection rates. Hence, these results suggest that PPV-2, PPV-3, PPV-6 and TTSuV-1a are significantly correlated with the probability of animals displaying the clinical signs of PRDC.

## Discussion

PRDC, a multifactorial disease whose aetiology is viral^2,16^, significantly impacts the productivity of the swine industry by increasing piglet morbidity and mortality^5,27^. In this study, 17 different viruses were identified in the samples collected from nasal swabs and sera from 26 PRDC-affected piglets, which shows that the viral communities in these animals were complex and diverse. A greater number of viral species were identified than those reported in previous studies^18,28^. Furthermore, differences in the viral flora were observed in the serum (16 viruses) and nasal swab (nine viruses) libraries from the diseased piglets, a finding possibly related to the effectiveness of the different sampling methods at fully identifying the viral categories. This discrepancy has also been observed in dairy calves with respiratory diseases^29^. Furthermore, the tissue distribution of the viruses may differ over their infection periods. Seventeen distinct viral sequences were detected in the lung samples from the 26 piglets, suggesting that the lungs might be a useful reference organ for a sampling method aimed at identifying the viruses associated with PRDC by metagenomics, and we have previously used lung samples to identify viromes with this technique. Nevertheless, a large proportion of the genome sequences from the host were present in the library, and few viral sequences were detected in the lung samples. Therefore, more efficient techniques are needed for the removal of host nucleic acids from samples to allow greater recovery of the widest range of viral flora from the lungs of PRDC-affected piglets.

PCV-2, PRRSV, PRV and SIV are recognized by the global pig industry as the primary viral pathogens associated with PRDC^2,17^, but in the present study SIV sequences were not identified in the serum and nasal swab samples by metagenomics. The viral pathogens associated with PRDC differ significantly among farms, production sites, regions and countries^1,3,4,30^. Hence, developing an unbiased identification scheme for viral communities is essential for implementing effective control regimens against PRDC on farms. One feature of PRDC is its association with co-infections containing multiple viruses^2,18^. In the present study, we identified at least 17 different viruses by PCR in the 26 piglets with PRDC. We found evidence of co-infections with at least five different viruses in the lung samples and, notably, co-infections with 11 different viruses were detected in one sample, which highlights the high prevalence of co-infections and diverse viral communities in porcine respiratory disease in China. We also found that PCV-2–PRRSV co-infections in the lung samples from the PRDC-affected piglets were significantly higher in number than those of the other viruses, which is in agreement with previous studies on PRDC^2,9^. However, the low abundance or complete absence of PCV-2 and PRRSV in the serum and nasal swabs, as determined by metagenomics, is likely to be related to the different sampling methods that were used and the viral distribution in these samples. Furthermore, co-infections with PRRSV significantly increase PCV-2 viremia and enhance the severity of piglet respiratory disease^31^. Therefore, these results suggest that both PCV-2 and PRRSV play important roles in the development and outcome of PRDC.

Our viral metagenomics analysis to characterize all the viruses present in the samples, combined with our assessment of the viral detection rate in biological samples from well-matched disease cases and healthy controls, could potentially provide a simple approach for studying complex infectious diseases^18^. In this study, when the distribution of the sequences arising from the 17 viruses in the serum samples was compared between the animals with PRDC and the clinically healthy controls, 6.81 and 4.09 viruses on average were detected per sample from the PRDC-affected animals and the clinically healthy controls, respectively. Shedding of six or more distinct viruses was also associated with PRDC infections. Co-infections with a large number of viruses might therefore overcome piglet innate immune defenses^1,32^. Single infections with PCV-2 or PRRSV, or co-infections with them both, had significantly higher detection rates than those for the other viruses in the PRDC-affected piglets, indicating that these two pathogens may be closely associated with the occurrence of PRDC in piglets. Interestingly, statistical analysis of the association between virus detection in the PRDC cases and the clinically healthy controls revealed that the novel viruses PPV-2, PPV-3, PPV-6 and TTSuV-1a were each associated with PRDC and might, therefore, be risk factors for contracting PRDC.

In recent years, the wide availability and usage of PCR-based methods and high-throughput sequencing have seen novel viruses discovered in piglets with PRDC, including PPVs and TTSuVs^10,27,33,34^. In the present study, most of the viruses identified in the diseased piglets by viral metagenomics were small linear and circular DNA viruses. Notably, the genome sequences from novel viral genotypes were successfully assembled and their biological identification suggests that viral evolution is far more complicated in piglets with PRDC than previously thought. Among the identified viruses, PPV-2, PPV-3, PPV-6 and TTSuV-1a are strongly associated with PRDC; however, the pathogenesis of these viruses is still unclear in piglets with respiratory disease. In clinical analyses, PPV-2, PPV-3, PPV-6 and TTSuV-1a often appear in co-infections with PCV-2 in diseased piglets with PRDC or postweaning multisystemic wasting syndrome^10,35–37^. Therefore, the ability to identify the novel viruses associated with PRDC is important for further studies on this disease, including the replication of our study using animals co-infected with other pathogens, animals from different herds and ultimately following herd vaccination to measure the impact of reducing the level of viral infections.

In conclusion, our study provides compelling evidence that the viral communities in serum and nasal swabs from piglets with PRDC are diverse and complex. Besides PCV-2, PRRSV and PRV, many additional viruses coexist in the piglet upper respiratory tract and serum of piglets. Through use of a case-control study, here, PPV-2, PPV-3, PPV-6 and TTSuV-1a were recognized as the candidate viruses associated with PRDC. Hence, our study provides a clearer picture than before of the viral communities in piglets with PRDC in Sichuan province, China, but further research is needed to determine the individual contribution these viruses make to PRDC in piglets.

## Materials and Methods

### Ethics statement

In this study, all the experiments were preformed according to the permit guidelines established by Southwest Minzu University, China. The experimental protocols were approved by the Animal Care and Use Committee of Southwest Minzu University and the Animal Disease Control Center of Sichuan province, China. The experimental animal certification number was SYXK2011-043.

### Sample collection

In the Sichuan province of China in 2016, 26 nursery piglets aged 1 to 2 months were collected from 11 intensive commercial farms where acute respiratory disease had broken out. Animals that exhibited the clinical signs of acute respiratory disease (i.e., fever, rhinorrhoea, cough, laboured breathing, depression and ocular discharge) were considered to be PRDC cases after assessment by a veterinarian. The piglets were adequately sedated with azaperone (Melone Biotechnology, Dalian, China) to avoid distress. The pathological alterations observed during necropsy in the lungs included diffuse bleeding, cyanotic appearance, interstitial thickening, swelling, and hyperemia. Prior to testing, the nasal swab samples from the piglets were placed into viral transport medium (VTM) at −80 °C. Serum and lung samples were stored at −80 °C before they were tested.

### Sample preparation and viral nucleic acid extraction

To avoid host gene contamination, serum and nasal swab samples were used to study the viral communities in 26 diseased piglets with PRDC. These samples were subjected to a series of pre-treatments based on a previously reported protocol^16^ before nucleic acids were extracted from them. Briefly, serum samples and VTM supernatants from the nasal swabs were collected from the piglets. Two pooled samples were assembled using 100 μL of each serum sample and the VTM supernatants from the nasal swab samples. The two pooled samples were each filtered through an unused 0.22-μm filter (Millipore, Billerica, MA, USA) to remove intact bacteria and large cellular debris. The filtrates were individually treated with 10 U of DNase and 1.5 μg of RNase enzymes (TaKaRa Biotechnology, Dalian, China) at 37 °C for 90 min to remove the unprotected nucleic acids. Total RNAs from both pooled samples were individually extracted using the QIAamp Viral RNA Mini Kit (QIAGEN, Hilden, Germany). The total RNAs obtained from each of the two samples were reverse-transcribed using SuperScript III reverse transcriptase (Invitrogen, Carlsbad, CA, USA) and random hexamers (Invitrogen) following the manufacturer’s protocol.

### Library construction and TruSeq Illumina sequencing

The cDNA amounts in both samples were determined by a Qubit Fluorometer (Life Technologies, Thermo Fisher, Waltham, MA, USA). The two cDNA preparations were used to individually construct two libraries according to the manufacturer’s instructions (TruSeq RNA Sample Preparation Kit, Illumina, San Diego, CA, USA). Briefly, the nucleic acids were ultrasonicated to generate fragments of less than 500-bp in length. The DNA fragments were end-repaired using T4 polynucleotide kinase, followed by adaptor ligation and loading onto the HiSeq 4000 (Illumina) for sequencing.

### Metagenomic data assembly and phylogenetic analysis

The Illumina-generated raw sequence reads were trimmed to remove the adaptor-related reads, duplicate reads, and porcine genomic sequences, and a 150 bp minimum length was selected. Reads that passed the data processing procedure were considered useful sequences, and these were aligned with the sequences in the NCBI nonredundant nucleic acid and protein databases using BLASTn and BLASTx, respectively. The taxonomies of the sequences with the best BLAST values were selected and used for further grouping analyses. The viral abundances were scanned by SOAP aligner software. In parallel, all useful sequence reads were subjected to *de novo* contig assembly using SOAP assembly software (http://soap.genomics.org.cn/) with the criterion of a 90% minimum overlap identity. Individual viral contigs from both pools were reassembled into a larger contig or genome sequence using high-level sequence identity criteria. The assembled viral nucleotide sequences and open reading frame-translated peptides were aligned against the nonredundant database with BLASTN and BLASTP algorithms to calculate their percentage identities. The global multiple nucleic acid sequence alignments were constructed for the full or near-full length viral genomic sequences using MUSCLE v3.7. Pairwise identity values and phylogenetic analyses were carried out using MEGA 7.0 software (https://www.megasoftware.net/). Phylogenetic trees were constructed using the maximum-likelihood method and the tree topology evaluation was based on 1000 bootstrap replicates.

### Virus detection rates in 26 piglets with PRDC

To detect the viral prevalence in the 26 nursery piglets with PRDC, the nucleic acids from the lung samples were extracted using the QIAamp Viral RNA Mini Kit (QIAGEN) and QIAamp Viral DNA Mini Kit (QIAGEN). The PCR primers (Table S1) were designed based on the reads from the Illumina sequencing run or the complete viral genomes from GenBank. The PCRs for each specific primer pair were performed using Quick Taq HS DyeMix at 1× concentration (Toyobo, Osaka, Japan) and primers each at 0.4 μM. DNA or cDNA (2 μL) from each lung sample was used in each reaction. PCRs were performed using the following conditions: a hot start of 95 °C for 5 min, followed by 35 cycles of 95 °C for 30 sec, 53 °C for 40 sec and 68 °C for 1 min. PCR products were gel-purified using the QIAquick gel extraction kit (QIAGEN). Both strands of the PCR products were sequenced at Sangon Biotech (Shanghai, China) using the same PCR primers.

### Virus detection in the serum samples from PRDC cases versus controls

An additional 74 serum samples from 36 PRDC cases and 38 location-matched clinically healthy control piglets of 1 to 2 months of age were collected from five other intensive commercial farms in Sichuan province (China) in 2017. Piglets, each with normal body temperature, dietary intake, respiration, nasal secretions and behaviour were regarded to be clinically healthy controls. Seven to nine serum samples from the diseased cases and clinically healthy controls were collected from each farm. The 17 different viruses identified in the 74 serum samples were investigated further using the primer pairs listed in Table S1. Fisher’s exact test was used to determine whether the virus detection rate was significantly higher in the PRDC cases than in the clinically healthy control animals. Odds ratios and their 95% confidence intervals were estimated for the association between each virus we identified and the probability of PRDC.

## Electronic supplementary material

Dataset 1, Dataset 2
