# Supplementary material for: Viral communities associated with porcine respiratory disease complex in intensive commercial farms in Sichuan province, China
Source: Sci Rep. 2018 Sep 6;8:13341. doi: 10.1038/s41598-018-31554-8 (PMC6127300; doi:10.1038/s41598-018-31554-8)
Supplement: Supplementary file 1 — Dataset 1, Dataset 2 [file 41598_2018_31554_MOESM1_ESM.pdf]

Viral communities associated with porcine respiratory disease complex in intensive commercial farms in Sichuan province, China

Running title: Viromes associated with PRDC

Sinan Qin[1], Wenqiang Ruan[1], Hua Yue[1,2,3*], Cheng Tang[1,2,3], Kelei Zhou[1], Bin Zhang[1,2,3*]

[1] College of Life Science and Technology, Southwest Minzu University, Chengdu 610041, China.

[2] Key laboratory of Ministry of Education and Sichuan Province for Qinghai-Tibetan Plateau Animal Genetic Resource Reservation and Utilization, Chengdu 610041, China.

[3] Animal Disease Prevention and Control Innovation Team in the Qinghai-Tibetan Plateau of State Ethnic Affairs Commission, Chengdu 610041, China.

Correspondence and requests for material should be addressed to B.Z. (email: binovy @sina.com) and H.Y. (email: yhua900@163.com)

Supplementary Table S1. Sequence information for the PCR primers used to detect the 17 different viruses in the 26 lung samples.

Virus	Primer sequence (5'→3')	Product length (bp)
PCV-2-F	GCGAGGAGGGTAATGAGG	275
PCV-2-R	CTCTGCAACGGTCACCAG	
PRRSV(NSP2)-F	GACACCTCCTTTGATTGG	493/797/887*
PRRSV(NSP2)-R	GAGAGGAIGCAGACAAATC	
PRV(gE)-F	GCTCTGCGTGCTGTGCTCC	346
PRV(gE)-R	GGGTCCATTCGTCACTTCCG	
PPV-2-F	AGCTCTGCGACAAGTGGG	563
PPV-2-R	GTCTACGGCCTGCAAGAA	
PPV-3-F	CCACGCCAAATCAAAGTC	514
PPV-3-R	CTCCCACTCCCATCCACT	
PPV-4-F	TATGTGGGCTGGGCAAGGAATGTC	416
PPV-4-R	GTTGCGGAATGCTATCAGGCTCTT	
PPV-5-F	ACACCTCCTGCGGCTTAT	959
PPV-5-R	GTGTAGCGATGTCCTGGC	
PPV-6-F	CTTTGGTGTAGAGGGCTTGA	650
PPV-6-R	CGGTTGTAGCAGGTCCAA	
PBoV-1-F	GTTTGGTGGGAAGAAGCA	491
PBoV-1-R	TTATCACCGCCGCAAGAT	
PBoV-3-F	CACAGGGGAAAGCATCGT	562
PBoV-3-R	CCACATTTGGTTCGGCAT	
PBoV-5-F	CGACTGCCAGAGCAAACT	465
PBoV-5-R	CCACAGCGACAACAAGGT	
TTSuV-1a-F	GGCTCAATTTGGCTCGCTTC	382
TTSuV-1a-R	CGATAGGCCCCTTGACTTC	
TTSuV-1b-F	CACCATACCCTGCCCCTT	413
TTSuV-1b-R	TGTCCTGTGACATTGAGTGAG	
PCMV-F	TGTGCGGTGAAGCAGTAT	284
PCMV-R	AGGCCTTACGGAGACCTT	
PKV-F	ACAGGGTGGATGAAGAACG	308
PKV-R	GAAGTTTGGAGCGAACCC	
PAstV-F	ACAGCAACCAAAGCCACA	372
PAstV-R	CACCACCACTCATTCACCTC	
PigSCV-F	TGCGTGGGCTATGTATCAA	398
PigSCV-R	CTGCGAGGACGTGCGTAA	

*493 bp (for NADC30-like PRRSV); 797 bp (for HP-PRRSV); 887 bp (for Classical PRRSV)

Supplementary Table S2. Viruses detected by PCR in 74 serum samples from PRDC-affected piglets and clinically healthy controls from five farms.

**Farm A**																
Virus	Asymptomatic samples							Rate*	Diseased samples							Rate*
	A1	A2	A3	A4	A5	A6	A7		B1	B2	B3	B4	B5	B6	B7	
PCV-2	+	+			+		+	4/7	+		+		+	+	+	5/7
PRRSV	+[2]	+[2]				+[3]		3/7	+[1]	+[2]	+[1]	+[1]	+[1]	+[1]	+[1]	7/7
PRV								0/7								0/7
PPV-2			+		+	+		3/7			+		+	+	+	4/7
PPV-3								0/7			+					1/7
PPV-4								0/7								0/7
PPV-5								0/7								0/7
PPV-6	+		+			+		3/7	+	+	+	+	+		+	6/7
PBoV-1								0/7								0/7
PBoV-3								0/7								0/7
PBoV-5								0/7				+				1/7
TTSuV-1a								0/7			+	+	+			3/7
TTSuV-1b		+						1/7				+		+	+	3/7
PCMV								0/7		+	+			+		3/7
PKV		+						1/7	+	+		+				3/7
PAstV		+	+	+	+	+		5/7	+	+	+	+	+		+	6/7
pigSCV		+						1/7								0/7

**Farm B**																	
Virus	Asymptomatic samples								Rate*	Diseased samples							Rate*
	A1	A2	A3	A4	A5	A6	A7	A8		B1	B2	B3	B4	B5	B6	B7	
PCV-2	+								1/8	+			+	+	+		4/7
PRRSV		+[2]							1/8	+[2]		+[3]		+[2]			3/7
PRV									0/8								0/7
PPV-2		+	+						2/8	+	+	+	+	+	+	+	7/7
PPV-3									0/8	+	+	+		+	+	+	6/7
PPV-4									0/8								0/7
PPV-5									0/7								0/7
PPV-6	+	+							2/8	+	+	+	+		+	+	6/7
PBoV-1									0/8								0/7
PBoV-3									0/8								0/7
PBoV-5									0/8				+				1/7
TTSuV-1a									0/8	+	+		+	+	+	+	6/7
TTSuV-1b									0/8			+	+		+	+	4/7
PCMV									08								0/7
PKV					+				1/8								0/7
PAstV			+		+		+		3/8	+	+	+	+			+	5/7
pigSCV									0/8								0/7

Farm C																
Virus	Asymptomatic samples							Rate*	Diseased samples							Rate*
	A1	A2	A3	A4	A5	A6	A7		B1	B2	B3	B4	B5	B6	B7	
PCV-2	+		+				+	3/7	+		+	+			+	4/7
PRRSV	+[2]		+[1,2]	+[2]		+[2,3]		4/7	+[2,3]	+[2]	+[2]	+[2]		+[2]	+[2]	6/7
PRV							+	1/7		+						1/7
PPV-2	+		+	+	+	+	+	6/7	+	+	+	+	+	+	+	7/7
PPV-3							+	1/7	+		+					1/7
PPV-4							+	1/7								0/7
PPV-5								0/7								0/7
PPV-6	+		+	+	+		+	5/7	+	+	+			+		4/7
PBoV-1								0/7								0/7
PBoV-3	+			+			+	3/7								0/7
PBoV-5	+							1/7								0/7
TTSuV-1a	+	+			+		+	4/7			+	+	+			3/7
TTSuV-1b	+	+			+	+		4/7	+			+		+		3/7
PCMV				+	+			2/7				+		+	+	3/7
PKV								0/7								0/7
PAstV	+	+	+	+	+			5/7	+	+	+	+	+		+	6/7
pigSCV								0/7			+			+		2/7

Farm D																
Virus	Asymptomatic samples							Rate*	Diseased samples							Rate*
	A1	A2	A3	A4	A5	A6	A7		B1	B2	B3	B4	B5	B6	B7	
PCV-2	+			+	+	+	+	5/7	+	+	+	+	+	+	+	7/7
PRRSV						+[1]		1/7	+[1]			+[1]	+[1]		+[2]	4/7
PRV								0/7								0/7
PPV-2	+			+		+		3/7		+	+	+	+	+	+	6/7
PPV-3				+	+			2/7		+	+	+	+	+		5/7
PPV-4								0/7								0/7
PPV-5					+			1/7			+					1/7
PPV-6				+				1/7	+	+	+	+	+	+		6/7
PBoV-1				+		+		2/7			+		+	+	+	4/7
PBoV-3								0/7		+				+		2/7
PBoV-5								0/7		+						1/7
TTSuV-1a				+			+	2/7	+		+		+	+	+	5/7
TTSuV-1b			+	+		+		3/7		+	+		+	+	+	5/7
PCMV								0/7		+		+				2/7
PKV	+	+					+	3/7			+			+	+	3/7
PAstV	+	+	+	+		+	+	6/7	+	+	+	+	+	+	+	7/7
pigSCV			+					1/7			+	+				2/7

Farm E																			
Virus	Asymptomatic samples									Rate*	Diseased samples								Rate*
	A 1	A 2	A 3	A 4	A 5	A 6	A 7	A 8	A 9		B 1	B 2	B 3	B 4	B 5	B 6	B 7	B 8	
PCV-2	+		+					+	+	4/9			+	+	+	+	+	+	6/8
PRRSV	+[1]				+[1]			+[1]		3/9	+[1]	+[1]	+[1]		+[1]	+[1]	+[1]		6/8
PRV										0/9									0/8
PPV-2		+	+	+	+			+	+	6/9	+	+	+			+	+	+	6/8
PPV-3								+	+	2/9	+		+	+	+	+		+	6/8
PPV-4		+	+							2/9				+					1/8
PPV-5				+	+					2/9				+					1/8
PPV-6	+	+	+	+	+	+	+	+	+	8/9	+	+	+	+	+	+	+	+	8/8
PBoV-1		+				+				1/9		+		+			+		3/8
PBoV-3		+								1/9		+							1/8
PBoV-5		+						+		2/9									0/8
TTSuV-1a				+					+	2/9									0/8
TTSuV-1b		+	+	+		+		+	+	6/9	+	+	+			+	+	+	6/8
PCMV	+			+		+	+	+	+	6/9	+	+	+	+	+	+	+	+	8/8
PKV										0/9									0/8
PAstV	+	+		+				+	+	5/9	+		+	+	+	+		+	6/8
pigSCV										0/9									0/8

1: Highly pathogenic PRRSV; 2: NADC 30-like PRRSV; 3: Classical PRRSV
*Detection rates of the 17 different viruses in serum samples.
